# Telemedicine During the COVID-19 Pandemic: Experiences From Western China

**DOI:** 10.2196/19577

**Published:** 2020-05-08

**Authors:** Zhen Hong, Nian Li, Dajiang Li, Junhua Li, Bing Li, Weixi Xiong, Lu Lu, Weimin Li, Dong Zhou

**Affiliations:** 1 Department of Neurology West China Hospital Sichuan University Chengdu China; 2 Department of Medical Affairs, West China Hospital, Sichuan University Chengdu China; 3 Medical Administration, Health Commission of Sichuan Province Chengdu China; 4 Department of Pulmonary and Critical Care Medicine West China Hospital Sichuan University Chengdu China

**Keywords:** COVID-19, coronavirus disease, medical education, pandemics, teleteaching, tele-education, telemedicine

## Abstract

Disasters and pandemics pose unique challenges to health care delivery. As health care resources continue to be stretched due to the increasing burden of the coronavirus disease (COVID-19) pandemic, telemedicine, including tele-education, may be an effective way to rationally allocate medical resources. During the COVID-19 pandemic, a multimodal telemedicine network in Sichuan Province in Western China was activated immediately after the first outbreak in January 2020. The network synergizes a newly established 5G service, a smartphone app, and an existing telemedicine system. Telemedicine was demonstrated to be feasible, acceptable, and effective in Western China, and allowed for significant improvements in health care outcomes. The success of telemedicine here may be a useful reference for other parts of the world.

## Introduction

Coronavirus disease (COVID-19) has been declared a pandemic by the World Health Organization (WHO), whose director-general expressed concerns about the “alarming spread and severity” as well as the “alarming levels of inaction” [1]. Although the outbreaks of severe acute respiratory syndrome (SARS) and Middle East respiratory syndrome (MERS) were associated with much higher respective case fatality rates (CFRs of 9.6% and 34.4%, respectively), the COVID-19 pandemic has led to more deaths due to the large number of individuals infected. Human-to-human transmission of the SARS-CoV-2 (severe acute respiratory syndrome coronavirus 2) virus has become the primary transmission route of the disease [2]. In this context, policymakers need to weigh the risks of inaction and pursue policies and strategies commensurate with the magnitude of the threat.

Pandemics and other public health emergencies typically lead to a surge in demand for medical care, which overwhelms local capabilities. Telemedicine can be broadly defined as the use of telecommunications technologies to provide medical information and services. The benefits of telemedicine in these situations have been well documented [3,4]. Telemedicine can support long-distance clinical care, education, and health administration, and its use has increased dramatically in the past decade.

There are a number of potential benefits to implementing telemedicine, including [5]:

- Improved access to information;
- Provision of care not previously deliverable;
- Improved access to services and increasing care delivery;
- Improved professional education;
- Quality control of screening programs;
- Reduced health care costs.

**Figure 1:**
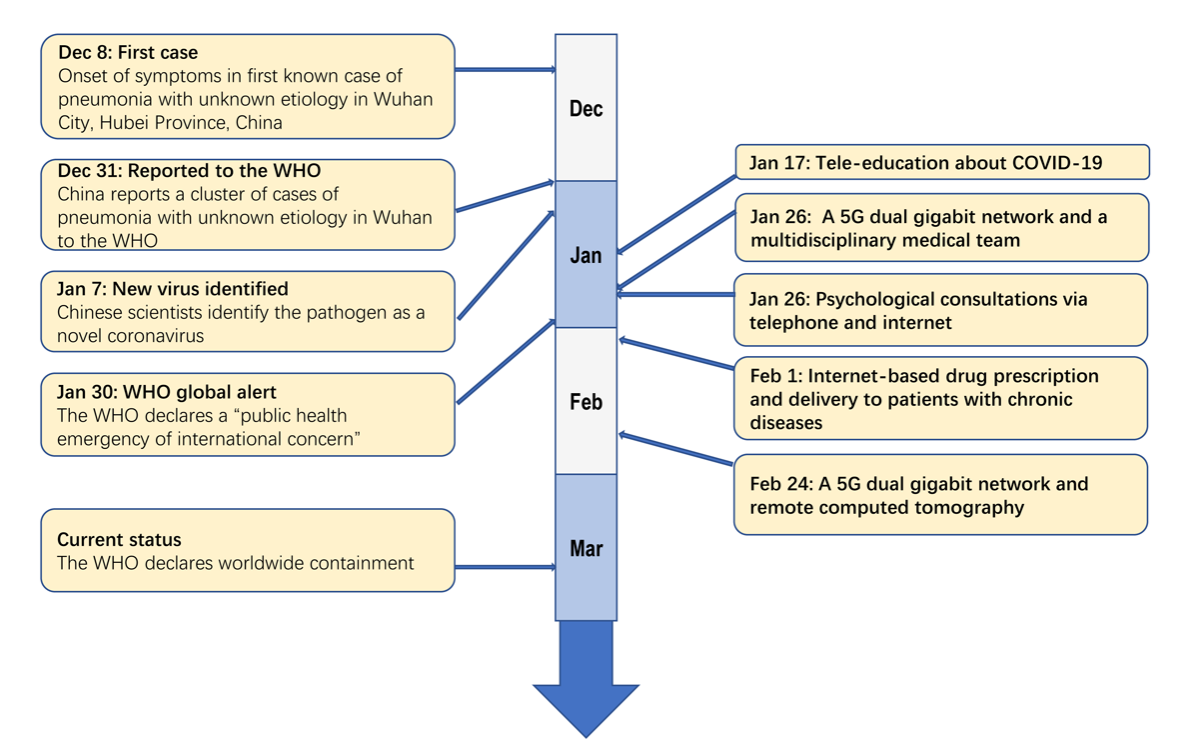
Progression of telemedicine in Western China during the coronavirus disease (COVID-19) pandemic. Left: timeline of COVID-19 events since the first case was reported on December 8, 2019. Right: timeline of telemedicine milestones in Sichuan Province of Western China during the COVID-19 pandemic. WHO: World Health Organization.

Western China does not have nearly as many economic resources or health care infrastructure as the eastern parts of the country. High-quality medical resources are concentrated in large- and medium-sized cities, and many county- and district-level hospitals face shortages of qualified personnel and inadequate technology for diagnosis and treatment [6]. Here, we share experiences of a multimodal telemedicine network in Sichuan Province in Western China during the COVID-19 pandemic. The network synergizes a newly established 5G service, a smartphone app, and an existing telemedicine system. The Sichuan telemedicine network was activated immediately after the first COVID-19 outbreak in January 2020 (Figure 1). Disaster response funds have been used to pay directly for telehealth care in the short term through a contingency contract with a telehealth network. It capitalized on the remote capabilities of staff and technology and likely played an important role during the pandemic.

## Tele-Education of Medical Staff About COVID-19

On January 17, 2020, an expert group for rapid response to COVID-19 was established in Sichuan Province; the experts provided tele-education to medical staff at local hospitals via remote consultation networks, portals, and smartphone apps. So far, more than 800,000 person-times have been devoted to training, helping to ensure the implementation of prevention and control measures against epidemics. Training topics include specimen collection methods, laboratory assays of nucleic acids, standardized diagnosis and treatment, prevention and control of hospital infections, personal protection, and medical waste disposal.

## A 5G Dual Gigabit Network and a Multidisciplinary Medical Team

**Figure 2:**
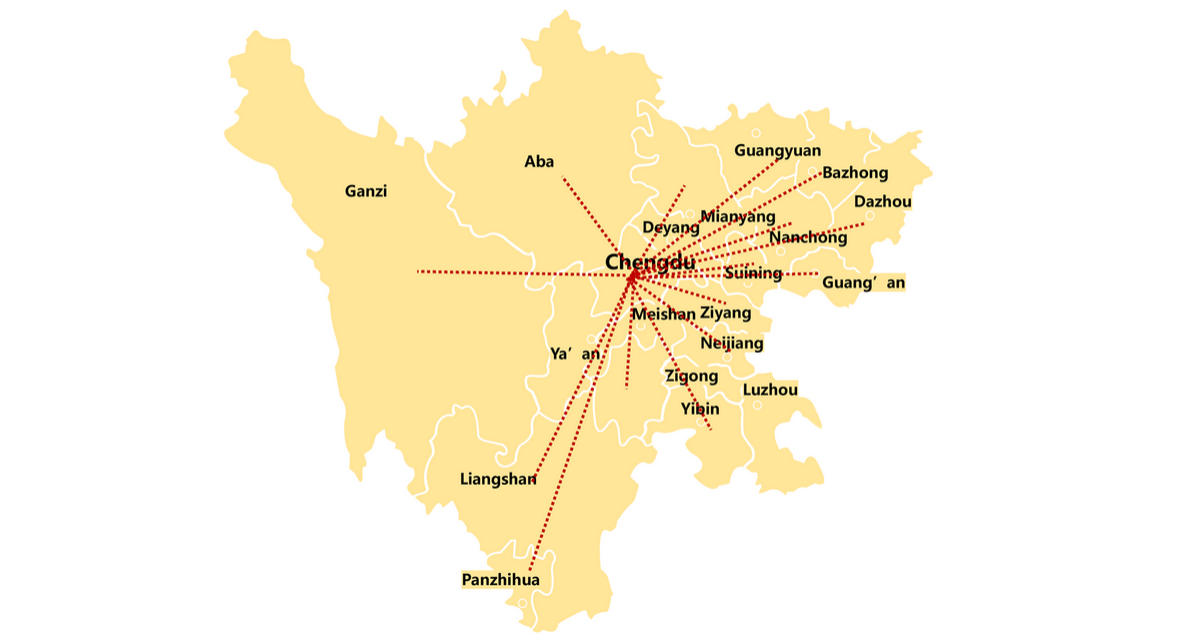
The 5G telemedicine network of Sichuan Province, China, established during the coronavirus disease (COVID-19) pandemic. This system makes use of the newly established China Telecom 5G Dual Gigabit infrastructure and currently covers all designated hospitals for COVID-19 of Sichuan Province (5 provincial-level, 24 municipal-level, and 179 county-level hospitals), with the West China Hospital of Sichuan University (WCHSU) as the central node. The median distance between a spoke hospital and WCHSU is 319 km (range 20 to 1191 km).

**Figure 3:**
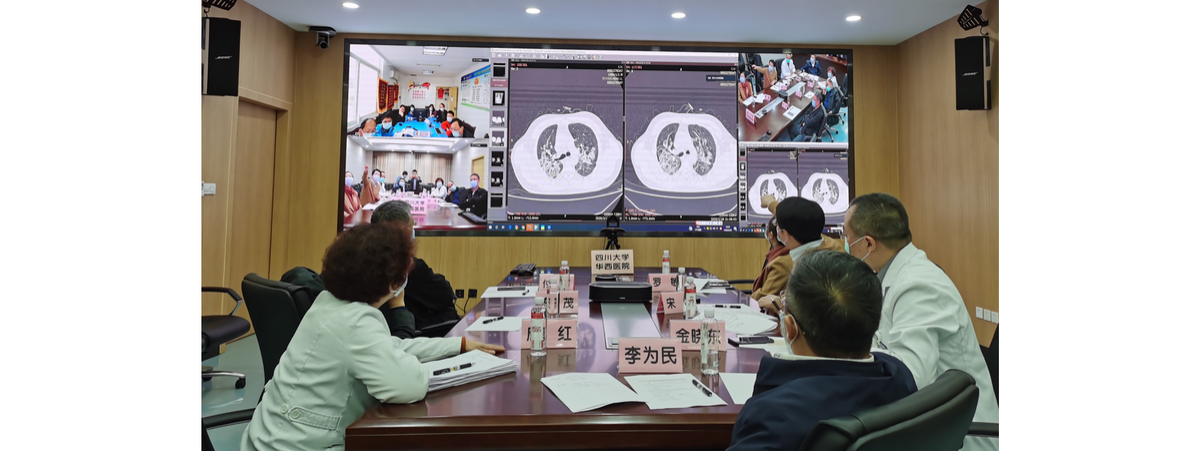
Web-based, real-time video telemedicine for consultations provided by a multidisciplinary team to deal with cases of coronavirus disease (COVID-19) in Western China.

On January 26, 2020, the West China Hospital of Sichuan University (WCHSU) launched a new web-based, real-time video telemedicine system for consultations provided by a multidisciplinary team to deal with COVID-19 cases (Figures 2 and 3). The network has focused on groups particularly vulnerable to severe symptoms of COVID-19, including the elderly, pregnant women, children, and patients with chronic health problems. From January 26 to March 12, 2020, 424 remote consultations were conducted for severe and critical COVID-19 patients, whose median age was 64 years (range 35 days to 87 years). Reasons for the consultations included adjustment of a patient’s antiviral therapy (75%), management of complications (68%), adjustment of respiratory therapy (55%), or confirmation of COVID-19 diagnosis (15%).

This new COVID-19 telemedicine system may increase diagnostic accuracy of difficult cases and improve treatment of severe or critical cases of COVID-19 for the large rural population of Western China at low cost; it has been highly praised by the WHO. This system may help explain why the CFR of COVID-19 is only 0.55% in Sichuan Province, which is much lower than the 4.64% in Hubei Province, China, and the mean rate of the world so far.

## A 5G Dual Gigabit Network and Remote Computed Tomography Scanning

On February 24, 2020, radiologists at WCHSU used the 5G Dual Gigabit network to remotely perform computed tomography (CT) on patients with COVID-19 at Ganzi People's Hospital in Ganzi County, Sichuan Province. To our knowledge, this is the first report of remote CT scanning during the COVID-19 pandemic. The experts at WCHSU were able to view the same images and data as the local clinicians at the same time, as well as remotely control the CT equipment at Ganzi People’s Hospital. On March 2, 2020, WCHSU clinicians remotely performed CT scanning of a COVID-19 patient at Huangzhou General Hospital in Huanggang City, Hubei Province. So far, 152 patients at hospitals in the telemedicine network have undergone remote CT scanning under the control and guidance of WCHSU physicians.

This marks the transition of telemedicine from the traditional “consultation” mode to a “practical operation” mode, ensuring high-quality CT even in areas with severe shortages of qualified technicians.

## COVID-19 Consultations Via Telephone and Internet

WCHSU officially opened a special COVID-19 telephone hotline and smartphone app for online consultations on January 26, 2020, through which medical staff offer free consultations and psychological interventions. By March 23, 2020, 9085 patients had received online consultations or interventions through the app, and 1094 patients had received consultations or interventions from 137 clinicians by telephone. Among these patients, 293 were screened for suspected COVID-19 and followed up.

## Internet-Based Drug Prescription and Delivery to Patients With Chronic Diseases

Hospitals are a potential source of COVID-19 cross-infection. To reduce accumulation of people in outpatient clinics during the pandemic, WCHSU and other major Chinese hospitals began to offer online consultations and internet-based drug prescription and delivery service for patients with common and chronic diseases since February 1, 2020. The internet-based services are offered through the hospitals’ websites, WeChat accounts, and widely used apps such as Huayitong. By March 23, 2020, 31,905 patients had received prescriptions or medicines through this service. This telemedicine service reduces the number of patient visits, eases overcrowding in outpatient centers, and allays worry among patients with chronic disease.

## Strengths, Limitations, and Future Directions

Pandemics pose unique challenges to health care delivery. As our experiences from Western China illustrate, although telemedicine will not solve all challenges, it can provide rapid access to specialists who are unavailable in person. With the rapid development of the internet and smartphone apps, telemedicine has transitioned to a multimodal paradigm, offering greater possibilities and convenience. The successes of telemedicine in Western China may provide a useful reference for other parts of the world. Prerequisites for success include sufficient financial resources, technological infrastructure, and overall arrangement of policymakers.

Telemedicine utilizes information and telecommunications technology to transfer medical information for diagnosis, therapy, and education. Although telemedicine clearly has a wide range of potential benefits, it also has some disadvantages. The main drawbacks of telemedicine are a breakdown in the relationship between health professionals and their patients; a breakdown in the relationship between health professionals; issues concerning the quality of health information; and organizational and bureaucratic difficulties [5]. In the future, with advancements in technology (eg, the application of 5G networks to improve the effect of video transmission) and improvement in the management experience of telemedicine by policymakers, the abovementioned limitations can be minimized, and telemedicine may become a sustainable, mainstream solution for both public health emergencies and routine medicine.

## Abbreviations

CFR: case fatality rate
COVID-19: coronavirus disease
CT: computed tomography
MERS: Middle East respiratory syndrome
SARS: severe acute respiratory syndrome
SARS-CoV-2: severe acute respiratory syndrome coronavirus 2
WCHSU: West China Hospital of Sichuan University
WHO: World Health Organization
